# Roles and Problems of Stroke Caregivers: A Qualitative Study in Yogyakarta, Indonesia

**DOI:** 10.12688/f1000research.52135.2

**Published:** 2022-01-31

**Authors:** Paryono Muhrodji, Hendrawan Dian Agung Wicaksono, Sekar Satiti, Laksono Trisnantoro, Ismail Setyopranoto, Amelia Nur Vidyanti

**Affiliations:** 1 Doctorate Program of Medical and Health Science, Faculty of Medicine, Public Health, and Nursing, Universitas Gadjah Mada, Yogyakarta, Indonesia, 55281, Indonesia; 2 Department of Neurology, Faculty of Medicine, Public Health, and Nursing, Universitas Gadjah Mada, Yogyakarta, Indonesia, 55281, Indonesia; 3 Center for Health Policy and Management, Faculty of Medicine, Public Health, and Nursing, Universitas Gadjah Mada, Yogyakarta, Indonesia, 55281, Indonesia

**Keywords:** caregiver, role, problem, homecare, stroke, post-stroke care

## Abstract

**Background**: Caregivers play a central role in post-stroke patients' care. However, the role of and problems managed by caregivers have not been widely studied, particularly in Indonesia. This study aims to explore the roles and problems of caregivers in post- stroke patients’ care.

**Method**: This was a qualitative study. Seven caregivers of post-stroke patients from the homecare clinic of Dr Sardjito General Hospital were purposely selected during January 2017 to June 2018. Focus group discussions were conducted to explore the roles and problems of caregiving.

**Results**: Themes related to caregivers’ roles were: connecting patients with medical personnel and other family members, maintaining patients’ health conditions by fulfilling basic needs and assisting rehabilitation, as well as maintaining patients’ psychological conditions by encouraging conversation, telling jokes, or recreation. On the other hand, themes related to caregivers’ problems were: lack of knowledge caused by education inadequacy, underappreciated and unconcerned family, suboptimal service including limited physiotherapy and pharmacy resource, unthorough administration, lack of communication, physical limitations, and burnout, as well as uncooperative patients.

**Conclusions: **Caregivers play essential roles as communicators and help to maintain patient's health conditions. Common problems are related to a lack of knowledge about strokes and a lack of attention from family. Further research to study the effects of these findings on the quality of life of both patient and their caregiver, as well as how to handle the caregiver issues should be investigated.

## Introduction

Caring for stroke survivors is a long-term experience that could influence a person’s quality of life. ^1^ In Indonesia, stroke has been the leading cause of death and disability for ten consecutive years (2009-2019). ^2^ Stroke, mainly affecting the elderly, may cause lifelong immobility, speech and communication problems, cognitive decline, urinary/fecal incontinence, and behavior changes that generally need long-term care. ^3, 4^ Therefore, stroke survivors become dependent on their caregivers, who take-on numerous roles and duties. ^3, 5^

Caregivers can be either formal or informal caregivers. ^6^ Formal care is delivered by professionals, such as nurses, physiotherapists, or speech and occupational therapists. Informal caregivers can be paid or unpaid. Unpaid informal caregivers are family members, friends, or relatives, meanwhile paid informal ones are domestic helpers or trained individuals. ^6^ Caregiving services can be provided in a home-based setting, a rehabilitation facility, or a nursing home. ^7^ In Asian population, the responsibility of caring the post-stroke patients mostly relies on family members, particularly the spouse or offspring who can provide caregiving in home-bound setting. ^8^

Caregivers play a central role in post-stroke patients’ care. A substantial amount of care, particularly to perform the activity of daily living, is essential for stroke survivors to improve their functional status. Therefore, caregivers are recommended to be involved in post-stroke patients care to improve the quality of patient management. ^7^

Currently, there are vast studies related to the experiences of caring for post-stroke patients. However, the role and problems managed by the caregiver while caring for post-stroke patients in Indonesia have not been widely studied. Accordingly, this study aims to explore the roles and problems of post-stroke caregivers in Indonesia.

## Methods

### Study design

This was a qualitative study to contextually explore a range of experiences and perspectives, not to seek an agreement. We used a grounded theory approach ^9^ to identify initial themes and categories which describe the roles and problems of caregivers for post-stroke patients.

### Sample and recruitment

We recruited caregivers from the homecare clinic of Dr Sardjito General Hospital during January 2017 to June 2018, using purposive sampling. Inclusion criteria were: (1) family caregivers or a domestic helper, who caregiving a post-stroke patient with any level of disability; and (2) providing care for ≥1 year. We sent a checklist of the eligible criteria to the homecare clinic. Staff at the homecare clinic subsequently provided us with potential participants’ phone numbers with their consent. We contacted the potential participants through telephone calls to invite them to the study. A focus group discussion was scheduled after receiving confirmation of their participation. A total of seven caregivers were included in this study. Two caregivers refused to participate in the study due to personal reasons.

### Data collection

Data were collected by conducting a focus group discussion (FGD) on 21 July 2019. The research team developed an interview guide which had been further transformed into several questions to be addressed in the discussion. The discussion was led by a moderator (the second author HDAW), assisted by a note-taker and an assistant who documented the discussion. The first author (PM, a senior neurologist) was also present during the discussion to supervise and guarantee that the discussion went effortlessly. The moderator (interviewer) was a male senior neurology resident who had basic training skills of FGD. He did not have any relationships with the participants and the participants knew him as a neurology resident who had interest in research related to stroke caregivers.

Participants’ demographic data were collected at the beginning of the discussion, along with that of their care recipients’ (post-stroke patients). These data included age, sex, education level, main occupation, relationship with post-stroke patient, and years of caregiving. The discussion began with a broad central question regarding the caregivers’ knowledge about stroke followed by probing and further questions related to roles and problems of caregivers. The discussion was held in a quiet and comfortable closed room (workplace) for approximately 120 minutes. It was carried out once and there were no repeat sessions.

### Data analysis

All discussions were tape-recorded and transcribed verbatim in Bahasa Indonesia. Data analysis began immediately after the discussion to identify ideas for generating categories. The semantic contents were separated and coded. Following a grounded theory approach, constant comparative analysis was used to compare data and open coding determined core categories. Identical codes were further grouped into categories to identify the main themes and subcategories. ^3^ QDA Miner Lite version 2.0.6 software was used to analyse the transcript and assist the coding process. The coding was further reviewed by two investigators (PM and HDAW) to establish that codes were data-driven. Any differences were discussed until consensus was reached. The full transcript was not returned to participants for comment.

### Ethical consideration

The study protocol received ethical approval from the Medical and Health Research Ethics Committee (Ethical approval reference number: KE/FK/0800/EC/2020) at the Faculty of Medicine, Public Health, and Nursing, Universitas Gadjah Mada, Yogyakarta, Indonesia.

### Consent

All participants were given a brief explanation of the research and gave written informed consent in accordance with the Declaration of Helsinki.

## Results

We enrolled seven caregivers; all subjects were female with a median age of 36 years (interquartile range [IQR] was 17). All had home-based responsibilities and half of them had less than 5 years of caregiving experience. These caregivers managed five post-acute stroke patients. Patients’ median age was 72 years (IQR was 14). Four (57.2%) of the caregivers had higher education while the other three (42.8%) had secondary education.

Five caregivers (71.4%) were full-time carers, while two others (28.6%) still had another job. Most of the caregivers (57,2%) were professional caregivers, while the other three (42.8%) were family members. One of the caregivers (14.2%) had been working as a caregiver for more than 10 years, two caregivers (28.6%) had been working for 5-10 years, and four others (57.2%) less than five years. The basic characteristics of the participants are depicted in Table 1.

**Table 1.** Characteristics of participants and post-stroke patients.

*Characteristics*		*Caregivers (n = 7)*		*Post-stroke patients (n = 5)*	
		**Median** **(min-max)**	**n (%)**	**Median** **(min-max)**	**n (%)**
***Age (years)***		36 (27-67)		72 (66-85)	
***Sex***	Male				1 (20%)
	Female		7 (100%)		4 (80%)
***Education level***	Higher education		4 (57.2%)		2 (40%)
	Secondary education		3 (42.8%)		3 (60%)
***Main occupation***	Caregiver		5 (71.4%)		
	Other		2 (28.6%)		
***Relationships with post-stroke patients***	Family		3 (42.8%)		
	Professional caregiver		4 (57.2%)		
***Years of caregiving***	<5 years		4 (57.2%)		
	5-10 years		2 (28.6%)		
	>10 years		1 (14.2%)		

After the discussion had been transcripted, 18 coding variables related to the roles of the caregiver for the post-stroke patient emerged from qualitative analysis. These 18 variables were reduced to six categories: communication, basic needs fulfillment, rehabilitation assistance, complication prevention and management, responsibility of patient’s condition, and maintenance of patient’s psychological condition. Furthermore, these six categories were reduced into three subthemes: communication, physical health, and patient’s psychology ( Table 2).

**Table 2.** Categories and themes of the role and problem of caregiver.

	*Themes*	*Subthemes*
***Caregivers’ roles***	**Communication**	Communication
	**Patients’ health**	Basic needs
		Rehabilitation
		Complications
		Responsibilities
	**Psychological**	Psychological
***Caregivers’ problems***	**Knowledge**	Knowledge
	**Family support**	Family support
	**Service limitation**	Resources of limitation
	**Communication**	Lack of communication with medical personnel
		Communication problems with patient
	**Physical and mental limitation**	Treatment refusal
		Fatigue (burnout)

A total of 28 codes which related to physical activity were identified which were then reduced into seven categories: knowledge, family support, limited resources and facilities, lack of communication with medical personnel, communication problem with patient, refusal to treatment, and fatigue (burnout). These seven categories are further reduced into five major subthemes: knowledge, family support, sub-optimal service, communication, as well as physical and mental limitation ( Table 2).

## Discussion

### Roles of caregiver: communication

Caregivers act as a communicator between the patient or family and medical personnel, or the patients and their family. Caregivers connect communication between the patient and people involved in the patient’s management including doctors, psychologists, nurses, and therapists. This role serves as an important aspect of patient care, and also be integrated as a guideline for adult stroke rehabilitation and recovery. ^7, 10^ Therefore, good communication skills are necessary for caregivers. ^11^

An example of this role can be seen in the following quote:

“ *... I have all the nurses’ contact numbers and mmm... what is it called... doctors’. And usually, if there is any problem... last night, for instance, she was using an urinary catheter and also a diaper. However, when I checked it, the diaper had been full with urine. In the morning, I sent text messages to a nurse and reported it: 'This blah blah blah, please check the catheter as it may be leaking...*” (Participant 6)

### Roles of caregiver: maintaining patients’ health

Caregivers as the people who daily assist patients’ activities, hold responsibility for patients’ health care. Caregivers provide basic needs fulfillment and rehabilitation processes, as well as prevention and management of complications. ^7, 11^

For providing basic need fulfillment, caregivers help patients with disabilities in need of self-care and mobility such as feeding, bathing or showering, toileting, dressing, grooming, walking, and transferring.

This quote below is an example from participant about this role:

“ *… within a day, our services are bathing, when it is eating time we feed them, and.. usually Mr M is simple, after bathing we feed him via PEG…*” (participant 4)

Caregivers also monitor the sleep pattern of their patients and help them to have good and adequate sleep. ^11, 12^

We can see an example of this role in this quote:

“ *… yes, I stand by for 24 hours. At night I sometimes sleep next to him, to supervise him …*” (participant 6)

During rehabilitation, caregivers also play a role in assiting rehabilitation processes by giving additional physiotherapy services at home. This is in accordance with the rehabilitation recommendations which state that families and caregivers are involved in determining the goals and implementation of post-stroke patient rehabilitation. ^10^

Role of caregiver in helping rehabilitation can be seen in this quote:

“ *… besides visiting physiotherapists in a hospital or inviting them to do home care services, in the morning and afternoon we usually have short additional physiotherapy session, only several minutes, just to stretch some muscles …*” (participant 1)

Furthermore, caregivers play essential roles in preventing and managing complications that may eventually develop. This role is in accordance with a previous study which found that caregivers can help reduce the incidence of post-stroke complications. ^13^ However, the study above used a formal training program for caregivers which was too impractical to be implemented in this study. The importance of training and education for caregivers is also supported by other studies that showed a reduction in complications through providing education programs to caregivers. ^14^

This role can be seen in the following quote:

“… *because my mother is ee .. her mobility is very limited, thus I need to feeding her using feeding tube. Other than that, what is it called? Catheter? Yes, a catheter! My mother is prone to urinary tract infection due to the catheter. Hence, sometimes if this infection develops, I have to... really have to do this, um ... to keep it clean.*.. "(participant 7)

Based on the above discussion, it can be observed that maintaining the good health of post-stroke patients is the responsibility of the caregiver. This is in accordance with prior studies which stated that there are major changes in the responsibilities of families caring for post-acute stroke patients. ^15– 17^

This can be elaborated with the quote from one of the interviewees below:

“... *this is my responsibility that she is completely dependent on us ... so we take care of her like 'because she is already completely dependent on us, so whatever happens to her, in my mind, it counts on us'...* ”(participant 6)

### Roles of caregivers: maintaining patients’ psychological condition

Caregivers spend a considerable amount of time engaging with patients and contribute to maintaining patients’ psychological conditions. ^14^ Based on the discussion, it was found that the caregivers help in maintaining patients’ psychological condition by telling interesting stories, joking, or doing recreational activities. This can be seen in the following statement:

“… *especially what we maintain is stabilitation of his mood. If he is happy, maybe mm... at least it doesn’t worsen his condition* …” (participant 5)

The role of caregivers in maintaining patients’ psychological health conditions is important in preventing depression in post-acute stroke patients. Depression is one of the most common complications of stroke. ^18^ Caregivers also have a great influence on the development of the patient's psychological condition and the prognosis. ^19^

### Caregivers’ problems: lack of knowledge

After conducting the FGD, it was found that one of the problems in post-stroke patient care is caregivers’ lack of knowledge. Caregiver illiteracy on stroke was clearly seen during FGD. When the caregivers were asked ‘what is stroke’, one of them answered:

“… *I actually don’t know a thing about stroke, the definition and how* …” (participant 6)

The other caregivers stated that they need to be equipped with some knowledge because for example, sometimes caregivers are involved in decision-making. However, they feel that they do not have sufficient knowledge to make a decision. This results in decision-making delay. The caregivers also feel that knowledge about medication and treatment is important. However, the information given to them by medical personnels is also limited. To overcome their lack of knowledge about stroke, they searched the information from other sources. However, the information obtained is said to be inaccurate, as in the statement below:

“… *sometimes it is confusing, you know. For example, in stroke management, some sources said it is okay to do acupuncture, some other said it’s not recommended, but... it's varying and confusing.*.. "(participant 2)

Lack of caregivers’ knowledge can be a weak point in post-stroke patients care. There have been many studies which stated that one of the important modalities that a caregiver must have is knowledge. ^7, 20, 21^ In addition, it is recommended that the process of decision-making and treatment planning should involve both the family and the caregiver. ^7, 22^

### Caregivers’ problems: underappreciated and unconcerned family

During the discussion, it was found that the family was not prepared for an emergency situation. This can delay early patient management. Meanwhile, some interviewees, domestic helpers being as informal caregivers, did not dare to take action before instruction had been given or a decision was made by the family, even though the patient was in an emergency condition. This occurred because the caregiver was afraid of being blamed, which can be seen in the following statement:

“… *we are afraid that later we will be blamed. I’ve experienced that. It’s like ‘that’s to bold’, or in Javanese ‘kok wani-wanine (how dare you?)’, even though we already did our best.*.. ”(participant 3, a domestic helper)

Another caregiver said that she had been scolded when she encountered similar situation, like in the phrase below:

“ *Even got scolded*” (participant 3, a domestic helper)

Moreover, the family did not give enough appreciation to the caregivers.

“ *no respect from the family*” (participant 2, a domestic helper)

Lack of support by the family becomes a burden for the caregiver. Furthermore, the lack of family support in post-stroke patients care may reduce the quality of patient care. Family support has an important role in the patients’ quality of life. ^23^

### Caregivers’ problems: suboptimal service

The main obstacle felt by the family is the lack of personnel or facilities offered by the home care unit. The interviewees stated that due to limited physiotherapy facilities and resources, the interviewees sought additional personnel from other resources. In addition, there were also some difficulties in getting the prescribed medication.

We also found that results of laboratory test delivered to family or caregiver were taking too much time. There was also a case of mistaken identity of a test result which was commented on by saying:

“ *I think it could be a fatal mistake*” (participant 7)

They also expressed problems in unthorough administration. These experiences can be seen in the following quotes:

“... *I feel that the number of the physiotherapy sessions is not enough, but I know that they are really busy. We divide the resources, that's why we add from the third party.*..” (participant 5)

“... *sometimes I'm confused, for example, the doctor said, 'Ma'am, the lab result is good', 'which lab result, doc? I think it’s been a while since the last lab checks'* …” (participant 6)

“ *The administration, I think, still have many rooms for improvement … Some things have been claimed, have been written, but then written again* …”(participant 7)

Limited resources become a challenge itself because it raises the gap between the standard of care and real-world capacity of service, ranging from prevention to post-acute care. ^24^

### Caregivers’ problems: lack of communication

Perceptions that were obtained from the caregivers were that there was a lack of communication between medical personnel causing uncollaborative patient management. They stated that this caused the patient's treatment to be time-consuming. Poor communication is associated with less effective services. ^7^

The problems above can be seen in the following quote:

“ *… for example, the physiotherapist came and asked about the patient’s development, then I explained ‘Oh she is blah, blah, blah’, ‘Okay, I will report it to the doctor’. Later, the doctor will come empty-handed … seems like uninformed. That is what sometimes makes us disappointed …*” (participant 7)

Apart from communication problems with medical personnel, we also found that communication problems with patients became a burden for the caregiver. This is especially experienced with patients who have communication disorders. In long-term care setting, caregivers’ anxiety rates have decreased. Nonetheless, caregivers’ depression rates and burdens are still high. ^25^

This was an example which expressing this problem:

“ *…after her second stroke she really was deteriorated, we didn’t know whether she still recognized us or not because there were no response at all from her, she was only staring but it’s a blank stare…*” (participant 7)

### Caregivers’ problems: physical limitations and burnout

Based on the FGD, one of the obstacles in post-stroke patients care was patients’ refusal of treatment. The caregivers thought the patient's morale, pain, and burnout were the causes of the refusal. Apart from the patient side, caregivers also face physical and mental burnout.

Depression is one of the complications that often occurs in patients with a history of stroke. ^18^ Periodic examinations or screening related to depression in the patients and their caregivers by their doctor is recommended. ^7^ In addition, the deteriorating psychological condition of the patient is a heavy burden for the caregiver. ^20^ Anxiety and depression while caring the stroke patients are related to the caregivers’ burden. ^26^

Long working hours, quality of patient health, stroke severity, and level of patient dependency are factors associated with caregiver’s burnout. ^27^ Other studies have shown that a high need for care is also a factor that influences caregiver’s burnout. ^28^ Moreover, the emotional condition of the patient also affects caregiver’s burnout. ^29^ The description of this problem can be seen in the following quote:

"... *during speech therapy, she persistently refused the therapy. The therapy ... at that time, it was from ... the speech therapy from Sardjito Hospital. When the therapist came, she pretended to sleep, and later after the therapist had came home, she woke up. She constantly refused it, she kept looking away. 'Ma'am, the therapist is coming ...' she insisted she did not want it. For communication, she responded, but she refused the therapy.*.. "(participant 7)

This study has several limitations. First, the caregivers in this study were recruited from one medical center, and the sample size was small. Second, our findings might not be generalized to non-Asian countries as our participants were recruited in Indonesia which has collectivist backgrounds. ^30^ Third, we did not distribute the transcription to the caregivers for verification, thus it may lead to misinterpretation. Finally, all the caregivers in our study were female which may cause differences in caregiving experiences and lead to different roles and problems. Future study can be conducted with male caregivers to capture holistic understanding of caregivers’ role and problems.

## Conclusions

Caregivers play an essential role in connecting patients with medical personnel and other family members, as well as maintaining the patient's physical and psychological condition. Meanwhile, caregivers face the problems of lack of support from patients' family, lack of knowledge, suboptimal service, lack of communication and caregivers’ burnout. Further research to study the effects of these findings on the quality of life of both patient and their caregiver, as well as how to handle the caregiver issues should be investigated.

## Data availability statement

### Underlying data

Zenodo: Roles and problems of stroke caregivers: A qualitative study in Yogyakarta, Indonesia. https://doi.org/10.5281/zenodo.4716264. ^31^

The project contains the following underlying data:

• English Translated Transcript-Edited-Final.doc (an English translation of the focus group transcript).

## Reporting guidelines

Zenodo. Roles and problems of stroke caregivers: A qualitative study in Yogyakarta, Indonesia. https://doi.org/10.5281/zenodo.4729367.

The project contains the following reporting checklist:

• COREQ_checklist.pdf

Data are available under the terms of the Creative Commons Attribution 4.0 International license (CC-BY 4.0).

## Author contributions

Muhrodji P: writing-original draft preparation, conceptualization, methodology, formal analysis; Wicaksana HAD: data curation, investigation; Satiti S: supervision, validation; Trisnantoro L: conceptualization, supervision, validation; Setyopranoto I: conceptualization, supervision, validation; Vidyanti AN: visualization, validation, writing-review & editing.
