# Supplementary material for: Roles and Problems of Stroke Caregivers: A Qualitative Study in Yogyakarta, Indonesia
Source: F1000Res. 2022 Jan 31;10:380. Originally published 2021 May 13. [Version 2] doi: 10.12688/f1000research.52135.2 (PMC8822138; doi:10.12688/f1000research.52135.2)
Supplement: Peer review history

# Peer review: Reviewer response for version 2

**DOI:** 10.5256/f1000research.120742.r121718

**Published:** 2022-02-07

**Authors:** Thuan Duc Nguyen

**Affiliations:** 1 Department of Neurology, Military Hospital 103, Vietnam Military Medical University, Hanoi, Vietnam

I do not have any further comments to make.

Is the work clearly and accurately presented and does it cite the current literature?

Yes

If applicable, is the statistical analysis and its interpretation appropriate?

Yes

Are all the source data underlying the results available to ensure full reproducibility?

Yes

Is the study design appropriate and is the work technically sound?

Yes

Are the conclusions drawn adequately supported by the results?

Partly

Are sufficient details of methods and analysis provided to allow replication by others?

Yes

Reviewer Expertise:

Stroke, Clinical Neurology especially Pain, Movement disorders.

I confirm that I have read this submission and believe that I have an appropriate level of expertise to confirm that it is of an acceptable scientific standard.

# Peer review: Reviewer response for version 2

**DOI:** 10.5256/f1000research.120742.r121717

**Published:** 2022-02-03

**Authors:** Aznida Firzah Abdul Aziz

**Affiliations:** 1 Department of Family Medicine, Faculty of Medicine, Universiti Kebangsaan Malaysia Medical Centre, Kuala Lumpur, Malaysia

The authors have revised the manuscript well- and the results and discussion of themes are better organised.

 Note:

 The quantitative results can be improved i.e.,

- "..median age of 36 years (interquartile range [IQR] was 17)" can be written as "..median age of 36 (IQR 17) years."
- "Patients’ median age was 72 years (IQR was 14)" can be written as "Patients’ median age was 72 (IQR 14) years"

The Conclusion section usually answers the study objectives, i.e., for this study refers to "..aims to explore the roles and problems of post-stroke caregivers in Indonesia". Hence the conclusion should recommend that future studies address the problems identified in this study and how to overcome them. Example would be perhaps to explore a module designed for training professional caregivers - since the problems highlighted in the study were mainly salaried caregivers. And the module would be appropriately written to address the educational level of the caregivers - instructions in lay person language, how to communicate with patients' relatives, addressing stress issues in caregivers e.g. to consider respite care for caregivers such as days or hours off from caregiving.

Is the work clearly and accurately presented and does it cite the current literature?

Yes

If applicable, is the statistical analysis and its interpretation appropriate?

Not applicable

Are all the source data underlying the results available to ensure full reproducibility?

Yes

Is the study design appropriate and is the work technically sound?

Partly

Are the conclusions drawn adequately supported by the results?

Partly

Are sufficient details of methods and analysis provided to allow replication by others?

Yes

Reviewer Expertise:

Family Medicine, Health system Research, Community Stroke & Economic evaluations

I confirm that I have read this submission and believe that I have an appropriate level of expertise to confirm that it is of an acceptable scientific standard.

# Peer review: Reviewer response for version 1

**DOI:** 10.5256/f1000research.55371.r94920

**Published:** 2021-10-29

**Authors:** Thuan Duc Nguyen

**Affiliations:** 1 Department of Neurology, Military Hospital 103, Vietnam Military Medical University, Hanoi, Vietnam

Stroke is the leading cause of disability worldwide and has become a socioeconomic burden for our society. Caring for stroke survivors can play a crucial role in their continuous recovery in which caregiver is indispensable to these activities. The study mentioned roles and problems of post-stroke caregivers, which is meaningful in clinical practice. The manuscript is well-written, presents the research contents in detail as well as analyzes, synthesizes and draws out results consistent with the research objectives. However, there are a few points the authors also need to pay attention to:

1. Data collection needs to be very accurate and avoid interference because the data here are completely subjective information of the research object. For example, participant 6 said " *…yes, I stand by for 24 hours. At night I sometimes sleep next to him, to supervise him…*" - Is it really accurate to be with the patient 24 hours a day? When will the caregiver sleep?
2. The study sample size is too small to draw general representative results. A very important problem related to caregiver is the lack of sympathy, sharing and love for the patients. They take care of patients after stroke often for the sake of salary and purely for responsibility. Is this problem encountered in this study?
3. Of the caregiver's roles and problems, which one is the most important? Can the authors rank the importance of those roles and issues?
4. The research objective only mentions the roles and problems of caregivers, so the conclusion should only focus on this point, not giving the opinion *"Understanding the roles and problems of caregivers may* *help facilitate better management and increase the quality of life for both patients and their caregiver* *”. *The author can suggest and suggest some points for further research in the future, which is more reasonable.

I hope the authors can edit, supplement and add their own discussion to make the study even more complete. This is really an interesting study.

Is the work clearly and accurately presented and does it cite the current literature?

Yes

If applicable, is the statistical analysis and its interpretation appropriate?

Yes

Are all the source data underlying the results available to ensure full reproducibility?

Yes

Is the study design appropriate and is the work technically sound?

Yes

Are the conclusions drawn adequately supported by the results?

Partly

Are sufficient details of methods and analysis provided to allow replication by others?

Yes

Reviewer Expertise:

Stroke, Clinical Neurology especially Pain, Movement disorders.

I confirm that I have read this submission and believe that I have an appropriate level of expertise to confirm that it is of an acceptable scientific standard, however I have significant reservations, as outlined above.

# Response

**Published:** 2022-01-27

**Authors:** Amelia Nur Vidyanti

**Affiliations:** Universitas Gadjah Mada, Indonesia

**Title: Roles and Problems of Stroke Caregivers: A Qualitative Study in Yogyakarta, Indonesia.**

***Response to reviewer 2***

*We wish to thank you all for your constructive comments in this review. Your comments provided valuable insights to refine its contents.*

Comments

 Stroke is the leading cause of disability worldwide and has become a socioeconomic burden for our society. Caring for stroke survivors can play a crucial role in their continuous recovery in which caregiver is indispensable to these activities. The study mentioned roles and problems of post-stroke caregivers, which is meaningful in clinical practice. ****The manuscript is well-written, presents the research contents in detail as well as analyzes, synthesizes and draws out results consistent with the research objectives. However, there are a few points the authors also need to pay attention to:

- Data collection needs to be very accurate and avoid interference because the data here are completely subjective information of the research object. For example, participant 6 said " *…yes, I stand by for 24 hours. At night I sometimes sleep next to him, to supervise him…*" - Is it really accurate to be with the patient 24 hours a day? When will the caregiver sleep?

**Response:** Thank you for your question. To clarify this quote, this statement from the participant was to depict that her duty was to standby for 24 hours. Of course, she did not wake up all day for 24 hours for monitoring and helping her patient. However, she meant that she was standby to caring her patient.

- The study sample size is too small to draw general representative results. A very important problem related to caregiver is the lack of sympathy, sharing and love for the patients. They take care of patients after stroke often for the sake of salary and purely for responsibility. Is this problem encountered in this study?

**Response:** The caregivers’ problem we found in the present study is not lack of sympathy, sharing and love for the patient, but more to lack of respect between caregivers and (other) family members. The different findings may be due to different definitions of caregivers in Asian population. We mentioned this in the revised version in line 64-72. In Asian population, the responsibility of caring the post-stroke patients mostly relies on family members, particularly the spouse or offspring who can provide caregiving in home-bound setting.

- Of the caregiver's roles and problems, which one is the most important? Can the authors rank the importance of those roles and issues?

**Response:** Thank you for your question, but we’re afraid that we cannot rank these roles and problems as we did not collect any quantitative data about these issues. We aim to identify the roles and problems of caregiving qualitatively.

- The research objective only mentions the roles and problems of caregivers, so the conclusion should only focus on this point, not giving the opinion *"Understanding the roles and problems of caregivers may help facilitate better management and increase the quality of life for both patients and their caregiver”. *The author can suggest and suggest some points for further research in the future, which is more reasonable.

**Response:** We would like to refine our conclusions. We agree that by understanding roles and problems of caregiver does not automatically improve post-acute care of stroke patient. Here is the revised conclusion:

**Conclusions**

 Caregivers play an essential role in connecting patients with medical personnel and other family members, as well as maintaining the patient's physical and psychological condition. Meanwhile, the problems faced by caregivers face the problems of were lack of support from patients' family, lack of knowledge, suboptimal service, lack of communication and caregivers’ burnout. Further research to study the effects of these findings on the quality of life of both patient and their caregiver, as well as how to handle the caregiver issues should be investigated.[hd1] 

  [hd1] [hd1] Revised conclusion as reviewer 2 suggested.

# Peer review: Reviewer response for version 1

**DOI:** 10.5256/f1000research.55371.r94919

**Published:** 2021-10-05

**Authors:** Aznida Firzah Abdul Aziz

**Affiliations:** 1 Department of Family Medicine, Faculty of Medicine, Universiti Kebangsaan Malaysia Medical Centre, Kuala Lumpur, Malaysia

**Title: Roles and Problems of Stroke caregivers: A Qualitative Study in Yogyakarta, Indonesia.**

**Abstract**:

 Satisfactory.

**General impression:**

 This is a novel attempt to document the roles and problems of stroke caregivers in Indonesia. The methodology is sound, but the manuscript would benefit from a more detailed description and discussion of the subject matter. The study setting needs to be introduced in greater detail as caregivers’ roles are very much affected by healthcare system differences across the world. Post stroke care is very challenging particularly for the LMIC, and especially for countries in the ASEAN region. Caregiver definitions in the Asian perspective also differ from western literature - and this should be highlighted in the write up. The authors may refer to this article (which I was involved with) by Ozdalifah O *et al. *(2021) ^1^ for definitions of stroke caregivers.

 Overall, this manuscript has potential to be accepted after major revision/refinement.

**Introduction:**

 This section was too brief (2 paragraphs) and did not highlight the caregiver situation in Indonesia (i.e. what is the norm in post stroke care, and who constitutes as caregiver?). There is mention that caregivers are made up of family members and professional caregivers. Suggest to highlight and define what professional caregivers are (i.e. salaried professional nurse/ therapist/trained domestic helper etc.) and where were the caregivers located - is it in the patients’ own home or at a inpatient rehabilitation facility/ nursing home?

**Method**:

 Focus group discussion was conducted to explore the roles and problems of caregiving (only ONE FGD was conducted, as mentioned in the article).

**Sample recruitment:**

 "Inclusion criteria were: (1) family or professional caregivers, who caregiving a post-stroke patient with any level of disability; and (2) providing care for ≥1 year."

***Comment*:**

 The definition of professional caregivers is unclear. Do you mean professional caregivers as trained and/or were they salaried caregivers?

**Data analysis:**

 Why was the transcription not distributed to the caregivers for checking, verification? Is there a specific reason for this?

**Results:**

 All subjects (caregivers) were female, median age of 36 years (?IQR), patients’ median age was 72 years (?IQR).

**Discussion:**

***Roles of caregiver: Communication:***

 The areas where communication problems most likely to occur should be grouped, summarized and examples quoted accordingly. Suggest to include examples of communication between:

- Caregiver and patient;
- Caregiver and medical personnel i.e. doctor;
- Therapists and doctor.

***Role of caregiver: maintain patients’ health:***

 Similarly, the flow of this section can be improved by citing the quotes after each point. Please maintain a consistent format throughout the results section. Below are suggested rewrites:

 Paragraph 2:

- “For providing basic need fulfilment, caregivers help patients with disabilities in need of self-care and mobility such as feed, bathing, showering, toileting, dressing, grooming, walking and transferring…”
- “Caregivers also monitor the sleep pattern of their patients and help them to have good and adequate sleep.”
- “….yes, I standby for 24 hours. At night I sometimes sleep next to him, to supervise him…’(participant 6, ?family/professional caregiver)

Paragraph 3.

- “During rehabilitation, caregivers play a role in assisting rehabilitation processes by giving additional physiotherapy services at home. This is in accordance with the rehabilitation recommendations that families and caregivers are involved in determining the goals and implementation of post-stroke rehabilitation. ^8^”
- “..besides visiting physiotherapists in a hospital or inviting them to do home care services, in the morning and afternoon we usually have short additional physiotherapy session, only several minutes, just to stretch some muscles….” (participant 17, family / professional caregiver).
- Caregivers’ problems: unappreciated and unconcerned family.
- “Moreover, the family did not give enough appreciation to the caregivers.
- “no respect from the family” (participant 2, ?professional/family caregiver)

**Conclusion:**

 This section should summarise and provide recommendations on how to handle the caregiver issues. E.g. identifying relevant information which should be passed on to the identified caregiver: information regarding stroke; overall management/rehabilitation plans; communication strategies between caregiver-therapist-doctor; therapist-caregiver; professional caregiver-family; caregiver respite issues (teaching caregivers on how to handle or cope with burnout, for professional caregivers to be given scheduled respite hours/days).

Is the work clearly and accurately presented and does it cite the current literature?

Yes

If applicable, is the statistical analysis and its interpretation appropriate?

Not applicable

Are all the source data underlying the results available to ensure full reproducibility?

Yes

Is the study design appropriate and is the work technically sound?

Partly

Are the conclusions drawn adequately supported by the results?

Partly

Are sufficient details of methods and analysis provided to allow replication by others?

Yes

Reviewer Expertise:

Family Medicine, Health system Research, Community Stroke & Economic evaluations

I confirm that I have read this submission and believe that I have an appropriate level of expertise to confirm that it is of an acceptable scientific standard, however I have significant reservations, as outlined above.

## References

[1] : Caregiver Depression Among Home-Bound Stroke Patients in an Urban Community. *Cureus* .2021; 10.7759/cureus.17948 10.7759/cureus.17948 PMC851432134660136

# Response

**Published:** 2022-01-27

**Authors:** Amelia Nur Vidyanti

**Affiliations:** Universitas Gadjah Mada, Indonesia

**Title: Roles and Problems of Stroke Caregivers: A Qualitative Study in Yogyakarta, Indonesia.**

***Response to reviewer*** **1.**

 We wish to thank you all for your constructive comments in this review. Your comments provided valuable insights to refine its contents. Because one of the objectives of this study is to widen our knowledge about caregivers’ roles and problems, especially in Indonesia, we would like to refine our paper. As you advise us to, more detail about this study setting and definitions about caregivers which enrolled in this study have been added.

*Comment 1*

**Introduction:**

 This section was too brief (2 paragraphs) and did not highlight the caregiver situation in Indonesia (i.e. what is the norm in post stroke care, and who constitutes as caregiver?). There is mention that caregivers are made up of family members and professional caregivers. Suggest to highlight and define what professional caregivers are (i.e. salaried professional nurse/ therapist/trained domestic helper etc.) and where were the caregivers located - is it in the patients’ own home or at a inpatient rehabilitation facility/ nursing home?

**Response:** We would like to thank you all for this comment. We agree that the differences in who cares for stroke patients (nurse or nonmedical-trained person) should be included in introduction to give our readers a bit of knowledge about how stroke patients are being cared in Indonesia. We have also added a reference as you suggest in refer to the definition of caregivers in Asian population.

**Revised text :**

 Caregivers can be either formal or informal caregivers ^6^. Formal care is delivered by professionals, such as nurses, physiotherapists, or speech and occupational therapists. Informal caregivers can be paid or unpaid. Unpaid informal caregivers are family members, friends, or relatives, meanwhile paid informal ones are domestic helpers or trained individuals ^6^. Caregiving services can be provided in a home-based setting, a rehabilitation facility, or a nursing home ^7^.In Asian population, the responsibility of caring the patients mostly relies on family members, particularly the spouse or offspring who can provide caregiving in home-bound setting ^8^. [ANV1] 

*Comment 2*

**Method:**

 Focus group discussion was conducted to explore the roles and problems of caregiving (only ONE FGD was conducted, as mentioned in the article).

**Sample recruitment:**

 "Inclusion criteria were: (1) family or professional caregivers, who caregiving a post-stroke patient with any level of disability; and (2) providing care for ≥1 year."

**Comment:**

 The definition of professional caregivers is unclear. Do you mean professional caregivers as trained and/or were they salaried caregivers?

**Response:** We thank for your valuable comment. In this study, we recruited only informal caregivers. We apologize by making a mistake in mentioning professional caregivers. Hence, we have revised the text as below:

 Inclusion criteria were: (1) family caregivers or a domestic helper[hd2] ,.

*Comment 3*

**Data analysis:**

 Why was the transcription not distributed to the caregivers for checking, verification? Is there a specific reason for this?

**Response:** There was no specific reason for not returning the transcript to respondents. We realize that it may lead to bias or misinterpretation, thus we put this issue in the limitation. We will take this point as a suggestion for improvement in further research.

*Comment 4*

**Results:**

 All subjects (caregivers) were female, median age of 36 years (?IQR), patients’ median age was 72 years (?IQR).

**Response:** Initially we only used median, minimum, and maximum number due to the sample size for this study is small. However, we have added the interquartile range as well in the revised version as below:

 We enrolled seven caregivers; all subjects were female with a median age of 36 years (interquartile range [IQR] was 17)[ANV3] . All had home-based responsibilities and half of them had less than 5 years of caregiving experience. These caregivers managed five post-acute stroke patients. Patients’ median age was 72 years (IQR was 14)[ANV4] . Four (57.2%) of the caregivers had higher education while the other three (42.8%) had secondary education.

*Comment 5*

**Discussion:**

***Roles of caregiver: Communication:***

 The areas where communication problems most likely to occur should be grouped, summarized and examples quoted accordingly. Suggest to include examples of communication between:

- Caregiver and patient;
- Caregiver and medical personnel i.e. doctor;
- Therapists and doctor.

**Response:** Communication problems had been found in caregivers’ problems’ section. We have rewritten this section as you suggest us to. Communication problems between therapists and doctors, while were very likely to occur, were not surfaced during FGD. There were some other themes that likely been caused by this particular problem (therapists and doctor’s communication), however we’re afraid that were just our assumption so we did not include this in our writing.

 Despite of this, we have re-organized the communication problems and added one more quote to describe related to communication problem between caregivers and the patients:

**Caregiver** **s’** **problems:** **lack of** **communication**

 Perceptions that were obtained from the caregivers were that there was a lack of communication between medical personnel causing uncollaborative patient management. They stated that this caused the patient's treatment was to be time-consuming. Poor communication is associated with less effective services ^7^.

 The problems above can be seen in the following quote:

 “ *…for example* *,* *the physiotherapist came and ask* *ed* *about the patient’s development, then I explained ‘Oh she is bla* *h,* *bla* *h,* *bla* *h* *’, ‘Okay, I will* *report it to the doctor* *’* *.* *L* *ater* *,* *the doctor will come empty-handed…seems* *like uninformed. That is what sometimes makes us disappointed…* ”(participant 7) [hd5] 

 Apart from communication problems with medical personnel, we also found that communication problems with patients became a burden for the caregiver. This is especially experienced with patients who have communication disorders. In long-term care setting, caregivers’ anxiety rates have decreased. Nonetheless, caregivers’ depression rates and burden s are still high ^25^.

 This was an example which expressing this problem:

 “… after her second stroke she really was deteriorated, we didn’t know whether she still recognized us or not because there were no response at all from her, she was only staring but it’s a blank stare…”[hd6] 

*Comment 6*

***Role of caregiver: maintain patients’ health:***

 Similarly, the flow of this section can be improved by citing the quotes after each point. Please maintain a consistent format throughout the results section. Below are suggested rewrites:

 Paragraph 2:

- “For providing basic need fulfilment, caregivers help patients with disabilities in need of self-care and mobility such as feed, bathing, showering, toileting, dressing, grooming, walking and transferring…”
- “Caregivers also monitor the sleep pattern of their patients and help them to have good and adequate sleep.”
- “….yes, I standby for 24 hours. At night I sometimes sleep next to him, to supervise him…’(participant 6, ?family/professional caregiver)

Paragraph 3.

- “During rehabilitation, caregivers play a role in assisting rehabilitation processes by giving additional physiotherapy services at home. This is in accordance with the rehabilitation recommendations that families and caregivers are involved in determining the goals and implementation of post-stroke rehabilitation. ^8^” 
- “..besides visiting physiotherapists in a hospital or inviting them to do home care services, in the morning and afternoon we usually have short additional physiotherapy session, only several minutes, just to stretch some muscles….” (participant 17, family / professional caregiver).

**Response:** We have re-organized this section by citing the quote after each point in order to improve the flow of writing.

**Roles of caregiver: maintaining patients’ health**

 Caregivers as the person people who daily assist patients’ activities, hold responsibility for patients’ health care. Caregivers provide basic needs fulfillment, and rehabilitation process es, as well as prevention and management of complications ^7,11 ^.

 For providing basic need fulfillment, caregivers help patients with disabilities in need of self-care and mobility such as feeding, bathing or showering, toileting, dressing, grooming, walking, and transferring.

 This quote below is an example from participant about this role:

 “ *…within a day, our services are bathing, when it is eating time we feed them, and.. usually Mr M is simple, after bathing we feed him via PEG…*” (participant 4)[hd7] 

 Caregivers[ANV8] also monitor the sleep pattern of the ir patients and help them to have good and adequate sleep ^11,12 ^.

 We can see this role in this quote:

 “…y *es, I stand by for 24 hours. At night I sometimes sleep next to him, to supervise him*…” (participant 6)[hd9] 

 During rehabilitation, caregivers also play a role in assisting rehabilitation process es by giving additional physiotherapy services at home. This is in accordance with the rehabilitation recommendations which stated that families and caregivers **are involved in determining the goals and implementation of post-stroke patient rehabilitation ^10^.

 Role of caregiver in helping rehabilitation can be seen in this quote:

 “… *besides visiting physiotherapists in a hospital or inviting them to do home care services, in the morning and afternoon we usually have short additional physiotherapy session, only several minutes, just to stretch some muscles*…” (participant 1)[hd10] 

 Furthermore, caregiver s plays essential role s to in prevent ing and managing complications that eventually may eventually develop. This role is in accordance with a previous study which found that caregivers can help reduce the incidence of post-stroke complications ^13^. However, the study above used a formal training program for caregivers which was rather too impractical to be implemented in this study. The importance of training or and education for caregivers is also supported by other studies that showed a reduction in complications through providing education programs to caregivers ^14 ^.

 This[ANV11] role can be seen in the following quote:

 “… *because my mother is ee .. her mobility is very limited, thus I need to feeding her using feeding tube. Other than that, what is it called? Catheter? Yes, a cathete* *r* *! My mother is prone to urinary tract infection due to the catheter. Hence, sometimes if this infection develops, I have to... really have to do this, um ... to keep it clean*... "(participant 7)

 Based on the above discussion, it can be observed that maintaining a the good health of post-stroke patients is the responsibility of the caregiver. This is in accordance with prior studies which stated that there are major changes in the responsibilities of families caring for post-acute stroke patients ^15-17 ^.

 This can be elaborated with the quote from one of the interviewees below:

 "... *this is my responsibility that she is completely dependent on us ... so we take care of her like 'because she is already completely dependent on us, so whatever happens to her, in my mind, it counts on us'...* "(participant 6)

*Comment 7*

- Caregivers’ problems: unappreciated and unconcerned family.
- “Moreover, the family did not give enough appreciation to the caregivers.
- “no respect from the family” (participant 2, ?professional/family caregiver)

**Response:** We appreciate reviewer’s comment related this matter. Caregivers’ problems related to unappreciated and unconcerned family were the problems faced by domestic helpers as the informal caregivers. Therefore, we have written additional explanation in the quotes of participant 2 and 3, as written in the revised text.

 During the discussion, it was found that the family was not prepared for an emergency situation. This can delay early patient management. Meanwhile, some interviewees, domestic helpers being as informal caregivers[ANV12] , did not dare to take action before instruction had been given or a decision was made by the family, even though the patient was in an emergency condition. This occurred because the caregiver was afraid of being blamed, which can be seen in the following statement:

 “… *we are afraid that later we will be blamed. I’ve experienced that. It’s like ‘that’s to bold’, or in Javanese ‘kok wani-wanine (how dare you?)’, even though we already did our* *best*... "(participant 3, a domestic helper[ANV13])

 Another caregiver said that she had been scolded when she encountered similar situation, like in the phrase below:

 " *E* *ven got scolded*" (participant 3, a domestic helper[ANV14])

 Moreover, the family did not give enough appreciation to the caregivers.

 " *no respect from the family*" (participant 2, a domestic helper[ANV15])

*Comment 8*

**Conclusion:**

 This section should summarize and provide recommendations on how to handle the caregiver issues. E.g. identifying relevant information which should be passed on to the identified caregiver: information regarding stroke; overall management/rehabilitation plans; communication strategies between caregiver-therapist-doctor; therapist-caregiver; professional caregiver-family; caregiver respite issues (teaching caregivers on how to handle or cope with burnout, for professional caregivers to be given scheduled respite hours/days).

**Response:** We appreciate your suggestions. However, we’re afraid that providing recommendation to handle these issues is out of scope of this paper. Maybe we can add some opinion to add some solution to these issues, but as the aim of this paper is to widen and deepen our knowledge about roles and problems of caregivers’, we decided not to add recommendation on how to handle the caregiver issues. Nevertheless, we have added a closing statement in the conclusion as below:

 Further research to study the effects of these findings on the quality of life of both patient and their caregiver, as well as how to handle the caregiver issues should be investigated.[hd16] 

 [ANV1] As reviewer 1 suggested, we add more explanation about caregivers and the setting/location of care provided

 [hd2] As reviewer 1 asked, we clarify the type of caregivers recruited

 [ANV3] As reviewer 1 suggested, we add the IQR

 [ANV4] As reviewer 1 suggested, we add the IQR

 [hd5] The quote has been organized and moved right after the first paragraph

 [hd6] As reviewer 1 suggested, we add one more quote related to communication problem between caregivers and the patients

 [hd7] We add one more quote to provide more example related to the role of caregivers for providing basic needs

 [ANV8] As reviewer 1 suggested, we have moved citing the quote after each point to improve the flow of writing

 [hd9] As reviewer 1 suggested, we have moved citing the quote after each point to improve the flow of writing

 [hd10] As reviewer 1 suggested, we have moved citing the quote after each point to improve the flow of writing

 [ANV11] As reviewer 1 suggested, we have moved citing the quote after each point to improve the flow of writing

 [ANV12] Additional explanation related to the status of caregivers as domestic helpers

 [ANV13] Additional explanation related to the status of caregivers as domestic helpers

 [ANV14] Additional explanation related to the status of caregivers as domestic helpers

 [ANV15] Additional explanation related to the status of caregivers as domestic helpers

 [hd16] Usulan revisi kesimpulan sesuai dengan saran reviewer 2
